# Women's fertility and vitamin D: Could hypovitaminosis D biomarkers correlate with the disease, and explain the unexplained female factor infertility?

**DOI:** 10.4314/ahs.v24i3.30

**Published:** 2024-09

**Authors:** Ibrahim A Albahlol, Ahmed Baker Alshaikh, Abdulrahman H Almaeen, Abdulrahman A Alduraywish, Umar Farooq Dar, Tarek H El-Metwally

**Affiliations:** 1 Departments of Obstetrics and Gynecology; 2 Pathology; 3 Internal Medicine; 4 Community and Family Medicine, College of Medicine, Jouf University, Sakaka, Saudi Arabia; 5 Department of Medical Biochemistry, Faculty of Medicine, Assiut University, Assiut, Egypt

**Keywords:** Female infertility, Polycystic ovary syndrome, unexplained infertility, Vitamin D, 25-Hydroxy-cholecalceferol, 1; 25-Dihydroxyl-cholecalceferol

## Abstract

**Objective:**

Vitamin D (Vit D) deficiency correlates women reproductive pathophysiology. We analyzed Vit D deficiency biomarkers in infertile women.

**Patients & method:**

This case-control study enrolled 80 infertile women polycystic ovary syndrome (PCOS), and other etiologies; anovulation and unexplained and 25 controls. Serum calcidiol and calcitriol were determined by ELISA and their direct ratio was calculated.

**Results:**

72% of controls and 92.5% of patients had Vit D deficiency/insufficiency (calcidiol mean ± SDM of 33.50±22.10 vs. 20.26±5.226 ng/mL and an AUC of 0.808±0.048). Calcitriol had an AUC of 0.909±0.031 that more effectively distinguished patients and etiologies (53.49±23.30 pg/mL) from controls (114.0±43.20 pg/mL; P<0.001). 4 other etiology cases and 17 controls had calcitriol levels ≥100 pg/mL. 64% of controls (4.090±0.020) and 16.25% of patients [2.634±0.855, P<0.04; 5 PCOS (3 primary/2 secondary), 3 secondary unexplained, and 5 others (one primary tubal, one primary/one secondary peritoneal, one primary/one cervical and one primary tubal had a normal ratio ≥3.333 at an AUC of 0.740±0.065. All biomarkers revealed patient levels ~50% lower than controls; lowest in PCOS and unexplained etiologies.

**Conclusion:**

Vit D levels are significantly reduced in infertile women; lowest in PCOS and unexplained etiology, for all biomarkers, where calcitriol was the optimal predictor of both infertility and etiology.

## Introduction

Female infertility places a substantial psychological, social, medical and economic burden on the couple and community. Over 70% of female infertility cases results from low ovarian reserve or dysfunction, issues with the uterus and cervix, tubal issues, and endometriosis and pelvic adhesions. However, 30% of cases are of unknown etiology 1,2. Vitamin D deficiency is prevalent in reproductive age women, and seasonal reproductive capacity may be in part a consequence of variations in Vit D levels. However, the literature to date is inconclusive and does not confirm a role for Vit D in female infertility 3-6. The total fertility rate in Saudi Arabia has dropped from 7.175 in 1950 to 2.148 in 2023; with a steady decline starting from 1980 (https://www.macrotrends.net/countries/SAU/saudi-arabia/fertility-rate, accessed May 20, 2023). To the Saudi ministry of health, causes of women infertility include ovulation disorders, polycystic ovary syndrome (PCOS), amenorrhea, pituitary dysfunction, endometriosis, fallopian tube blockage due to salpingitis as in pelvic inflammatory disease, previous infection with gonorrhea and chlamydia, or any abdominal surgery, and uterine abnormalities such as uterine fibroids (https://www.moh.gov.sa/en/HealthAwareness/Educational-Content/wh/Pages/036.aspx, accessed May 20, 2023). In a Saudi nation-wide representative sample of 15810 general population women aged 18-44 years, rate of involuntary infertility ranged from 25 to 50% within one year^7^,^8^. In Arar, Saudi Arabia, the prevalence of infertility among followed up women was 65.3%; 19.8% of them were primary and 80.2% were secondary infertility; ovulation defect constituted 24.6%, followed by the polycystic ovary (21.8%), tubal adhesions or obstruction (6.7%), endometriosis (3.2%), and uterine fibroid (3.0%) 9. In Najran, Saudi Arabia, 65% of followed up women infertility was primary and 35% was secondary infertility, with PCO disease as the most common (56%), followed by fibroids (22%), endometrial polyps (9%), adenomyosis (5%), hydrosalpinx (4%), congenital abnormality (2%), and other causes (1%) 10. Among Saudi women followed up in Riyadh city, 67.0% had secondary infertility, while 33.0% had primary infertility. Defective ovulation constituted 28.9%, tubal adhesion or obstruction was 7.4%, and male factors was 12.6% 11. To Khadawardi prevalence of infertility was 23.3% among followed up Saudi women, with endometriosis constituting 10.7% 12.

Vit D is a steroidal preprohormone with extensive immunomodulatory, anti-inflammatory, and metabolic functionality, including cell cycle control, insulin secretion, and anti-autoimmunity. The subsequent hydroxylation of Vit D to its prohormone 25-hydoxy-form (calcidiol) occurs in the liver and other tissues, and then further to the hormone form 1,25-dihydroxy-chole/ergo-calciferol form (calcitriol) in the kidney and other tissues (including epithelial and immune cells) is controlled by its own level and by various hormones, sterols and growth factors 4,5,13. Among 1000 genes controlled by Vit D receptor-mediated transcription and membrane-receptor signaling, those implicated in women infertility include insulin secretion and action, and expression of Homeobox 10, miR222, antimüllerian hormone, immunomodulatory and anti-inflammatory cytokines/chemokines, steroidogenesis, angiogenesis and antioxidant defense 14-20. Deficiency of Vit D, an essential micronutrient, is a global epidemic, as a consequence of low levels obtained by consumption of unfortified foods and insufficient production via conditional synthesis from 7-dehydocholesterol upon exposure of epidermal keratinocytes to UVB rays. Blood levels vary depending on a number of individual factors, including genetic polymorphisms, skin color and ethnicity, body mass index (BMI), and supplement use, as well as by environmental, life-style, food fortification policies, drug and pollutant exposure, and seasonal variations. Conditions that affect absorption, digestion and transport of the vitamin also lead to variations in blood levels. In the circulation, calcitriol is approximately 1000 times lower than calcidiol 4,5,13,21-23. Discrepancy among published literature considering Vit D role in female infertility may also originate from variance in the pre- and analytical factors.

The putative role for Vit D in female reproduction is indicated by uterine hypoplasia and anovulation in Vit D receptor (VDR) and 1α-hydroxylase knockout mice, (reviewed by Lerchbaum and Obermayer-Pietsch)^24^. Expression of HOXA10, required for multiple aspects of fertility - including the maintenance of a healthy pregnancy, is upregulated by Vit D. The VDR and metabolizing enzymes are expressed in female reproductive and endocrine tissues. Vit D deficiency is shown in the clinic and lab to result in poorer fertility and impaired functionality of the reproductive system, PCOS, endometriosis and pre-term birth, along with many systemic conditions that include increased insulin resistance^4^,^5^,^25^-^27^. Vit D controls the expression of aromatase and estrogen production, and increase sensitivity to FSH and human chorionic gonadotropin secretion, and estrogen and progesterone synthesis in human placenta 28. Vit D supplementation resulted in significant reductions in total testosterone, free androgen index, hirsutism, and C-reactive protein (CRP) levels, as well as a significant increase in sex hormone binding globulin and total antioxidant capacity 29,30. Serum calcidiol concentration significantly correlated with female infertility with insignificant difference in its level between primary and secondary causes 6. Low Vit D is correlated with poorer in vitro fertilization (IVF) outcomes, folliculogenesis and reduced ovarian reserves 5,27,31,32. In IVF, women with normal serum Vit D levels (comparable with follicular levels) have a significantly higher chance of euploid blastocyst production than deficient patients^33^. In healthy reproductive-aged Caucasian women, Vit D levels are significantly negatively correlated with multiple sex hormones 34. Polymorphisms in the Vit D binding protein (VDBP) that reduce its expression are associated with metabolic syndrome and low Vit D in patients and controls; however, this is not directly correlated with PCOS 35. In Pakistani women, one VDBP polymorphism, which activates 1a-hydroxylase, and inactivates 24-hydroxylase, exhibits an inverse correlation between Vit D levels and PCOS 36. Consequently, many fertility clinics advise screening for (generally as calcidiol levels) and supplementation of Vit D, most critical for women who are not at risk of ovarian hyper-stimulation but suffer from PCOS or similar. Supplementation of Vit D led to higher rates of positive pregnancy tests than in unsupplemented cases 4,5,25,27,37. Almost 96% of women in IVF treatment were Vit D deficient/insufficient, and this negatively correlates with duration of infertility and BMI 38.

Proposing an inverse relationship, herein we determine the correlation between Vit D biomarkers, calcidiol, calcitriol and their direct ratio, with multiple causes of infertility in a local population of healthy and infertile Saudi women.

## Patients and method

### Setting and Patients

This case-controlled study was conducted between September 12, 2018 and September 30, 2019 at Aljouf Maternity and Children's Hospital and College of Medicine, Jouf University, Sakaka, Saudi Arabia. The bioethical protocols (#6-16-4/40) were approved by committees at the university and Ministry of Health, and informed consent was provided by all participants included - in adherence to the provisions of the Declaration of Helsinki. Participants were excluded if they demonstrated unwillingness, have previous pelvic surgery, male factor infertility, or other co-morbidities include thyroid disorders and chronic conditions (i.e., liver, kidney, autoimmune diseases and diabetes). Patients prescribed glucocorticoids, antifungal, antiepileptic and antiretroviral drugs were also excluded, due to effects of these drugs for Vit D metabolism. Infertility (post-male factor exclusion) results in no conception after one year of unprotected intercourse 1. No prior pregnancy is termed primary infertility.

### Clinical Examination and Assessment

105 participants were included in the study, of which 25 were healthy (previously pregnant; aged 27.6 ± 5.3 years) controls and 80 were infertile (aged 27.76 ± 5.052 years with a duration of 3.538 ± 1.807 years). Of the infertile women, there were three groups based on etiology: 30 with PCOS, 27 with other etiologies (incorporating tubal n = 6, tubo-peritoneal n = 5, cervical n = 6 and uterine n = 5 issues and anovulation n = 5), and 23 of unexplained etiology. Unexplained classification only occurred after full investigations, including laparoscopy. These groupings were further defined by primary (n = 35)/secondary (n = 45) infertility status. A complete medical history was taken for all patients, and weight, height and vital signs were recorded and a complete examination was conducted systematically. Following laboratory and infertility investigations, individuals with abnormal findings were excluded from the study.

### Blood Sampling and Investigations

Serum was recovered from 5 mL peripheral blood samples via clotting and centrifugation. Aliquots were stored at -80°C. Calcidiol (ng/mL; cat# SL2762Hu) and calcitriol (pg/mL; cat# SL2845Hu) were measured using ELISA kits in accordance with instructions (Sunlong Biotech Co. Ltd., Zhejiang, China). Participants were grouped according to clinically designated levels, from severely deficient < 10 ng/mL to high/toxic at 50/> 80 ng/mL 39-42. However, the clinical cutoff levels do not pertain to female infertility. We also determined the direct calcitriol/calcidiol ratio for all participants.

### Data Analysis Procedure

Data analysis used SPSS (Statistics package for social sciences, Version 23.0, IBM Corp, Armonk, NY) and Prism-7.0 Package (GraphPad Software, San Diego, CA). Qualitative statistics were described as frequencies/percentages, while quantitative as mean ± standard deviation (SD)/median ± interquartile range (IQR). Variable distribution was determined by the Kolmogorov-Sminov test, non-normal variable distribution was identified by a significant p-value. Kruskal-Wallis test was used followed by Mann-Whitney test for multiple comparisons of Vit D levels in the groups. The area under the curve (AUC) was determined by ROC for both biomarkers and their ratio to discriminate the cases and controls, where higher AUC represent greater sensitivity and specificity for the biomarker. A p-value of ≤0.05 at a confidence level of 95% was considered significant.

## Results

Comparisons of the characteristics and investigations among participants (Table 1)

**Table 1:** Variations in serum calcidiol, calcitriol and their ratio as biomarkers for vitamin D status when investigating infertile (n = 80) and fertile (n = 25) women, described as range, mean ± SDM and number

Parameter		Group/Subgroup		Range	Mean ± SDM	n
Age, Years	Controls			19-38	27.600 ± 5.300	25
	Infertile	All		20-42	27.760 ± 5.052	80
		PCOS		20-34	25.500 ± 3.203	30
			PCOS-Primary	21-34	25.380 ± 3.074	21
			PCOS-Secondary	20-33	25.780 ± 3.667	9
		Unexplained infertility		22-42	29.780 ± 5.815	23
			Unexplained-Primary	22-32	25.140 ±3.532	7
			Unexplained-Secondary	25-43	31.810 ± 5.492	16
		Other infertility		21-40	28.560 ± 5.228	27
			Others-Primary	21-27	25.000 ± 2.517	7
			Others-Secondary	24-40	29.800 ± 5.396	20
BMI, kg/m^2^	Controls			18.2-25.2	22.200 ± 2.000	25
	Infertile	All		17.8-30.4	24.390 ± 2.804	80
		PCOS		24.2-30.4	26.820 ± 1.736	30
			PCOS-Primary	24.2-30.4	27.010 ± 1.962	21
			PCOS-Secondary	24.8-27.6	26.370 ± 0.985	9
		Unexplained infertility		17.8-26.8	22.220 ± 2.427	23
			Unexplained-Primary	17.8-24.2	20.540 ± 2.435	7
			Unexplained-Secondary	18.4-26.8	22.960 ± 2.093	16
		Other infertility		19.4-26.9	23.530 ± 1.951	27
			Others-Primary	22.6-26.1	24.270 ± 1.424	7
			Others-Secondary	19.4-26.9	23.270 ± 2.072	20
Gravidity	Controls			1-9	3.880 ± 2.030	25
	Infertile	All		0-4	0.989 ± 1.097	80
		PCOS		0-2	0.367 ± 0.615	30
			PCOS-Primary		0 ± 0	21
			PCOS-Secondary	1-2	1.222 ± 0.441	9
		Unexplained infertility		0-3	1.261 ± 1.054	23
			Unexplained-Primary		0 ± 0	7
			Unexplained-Secondary	1-3	1.813 ± 0.750	16
		Other infertility		0-4	1.444 ± 1.251	27
			Others-Primary		0 ± 0	7
			Others-Secondary	1-4	1.950 ± 1.050	20
Parity	Controls			0-8	3.520 ± 1.980	25
	Infertile	All		0-3	0.675 ± 0.925	80
		PCOS		0-1	0.167 ± 0.379	30
			PCOS-Primary		0 ± 0	21
			PCOS-Secondary	0-1	0.556 ± 0.527	9
		Unexplained infertility		0-3	1.087 ± 1.041	23
			Unexplained-Primary		0 ± 0	7
			Unexplained-Secondary	0-3	1.563 ± 0.892	16
		Other infertility		0-3	0.889 ± 1.013	27
			Others-Primary	0	0 ± 0	7
			Others-Secondary	0-3	1.2 ± 1.005	20
Disease Duration, Years	Controls					25
	Infertile	All		1-11	3.538 ± 1.807	80
		PCOS		1-5	2.800 ± 1.157	30
			PCOS-Primary	1-5	2.667 ± 1.111	21
			PCOS-Secondary	1-5	3.111 ± 1.269	9
		Unexplained infertility		2-9	4.348 ± 2.102	23
			Unexplained-Primary	2-5	3.286 ± 0.951	7
			Unexplained-Secondary	2-9	4.813 ± 2.316	16
		Other infertility		2-11	3.667 ± 1.861	27
			Others-Primary	2-5	3.286 ± 1.113	7
			Others-Secondary	2-11	3.800 ± 2.067	20
Calcidiol, ng/mL	Controls			16.1-92.5	33.500 ± 22.100	25
	Infertile	All		13.1-34.4	20.260 ± 5.226	80
		PCOS		13.1-29.25	17.310 ± 3.944	30
			PCOS-Primary	13.1-21.8	16.760 ± 3.074	21
			PCOS-Secondary	13.22-29.25	18.600 ± 5.488	9
		Unexplained infertility		13.8-23.7	18.670 ± 2.816	23
			Unexplained-Primary	13.8-22.4	18.130 ± 3.425	7
			Unexplained-Secondary	13.8-23.7	18.900 ± 2.597	16
		Other infertility		17.99-34.4	24.900 ± 4.931	27
			Others-Primary	20.4-30.2	24.490 ± 3.229	7
			Others-Secondary	17.99-34.4	25.040 ± 5.467	20
Calcitriol, pg/mL	Controls			49.8-221	114.00 ± 43.200	25
	Infertile	All		30.2-122.2	53.490 ± 23.300	80
		PCOS		30.2-92.5	44.950 ± 17.500	30
			PCOS-Primary	30.7-92.2	43.520 ± 14.940	21
			PCOS-Secondary	30.2-82.2	48.270 ± 23.10	9
		Unexplained infertility		30.4-88.8	48.500 ± 15.640	23
			Unexplained-Primary	31.8-62.6	43.910 ± 9.423	7
			Unexplained-Secondary	30.4-88.8	50.510 ± 17.590	16
		Other infertility		33.2-122.2	67.230 ± 28.260	27
			Others-Primary	38.2-122.2	79.630 ± 31.460	7
			Others-Secondary	33.2-111.8	62.900 ± 26.530	20
Calcitriol/Calcidiol Ratio	Controls			1.14-9.96	4.090 ± 2.020	25
	Infertile	All		1.255-5.454	2.634 ± 0.855	80
		PCOS		1.255-5.456	2.616 ± 0.858	30
			PCOS-Primary	1.76-5.456	2.624 ± 0.851	21
			PCOS-Secondary	1.255-4.031	2.599 ± 0.926	9
		Unexplained infertility		1.65-4.311	2.612 ± 0.777	23
			Unexplained-Primary	1.893-2.953	2.454 ± 0.457	7
			Unexplained-Secondary	1.65-4.311	2.681 ± 0.886	16
		Other infertility		1.34-4.921	2.674 ± 0.942	27
			Others-Primary	1.675-4.921	3.221 ± 1.145	7
			Others-Secondary	1.34-4.175	2.482 ± 0.807	20

### Age

Participant age was not a significant factor between controls and infertile group/subgroups, with the exception of secondary unexplained infertility vs. PCOS (P < 0.01) and primary PCOS patients (P < 0.01).

### BMI

PCOS individuals exhibited highest BMIs, while unexplained infertility group were the lowest, specifically primary conditions. Controls vs. infertility group and subgroups were significantly different P < 0.01 and P < 0.001, respectively, as were the infertile group vs. PCOS (P < 0.001), primary PCOS (P < 0.001), unexplained (P < 0.01) and primary unexplained cases (P < 0.01). Further, PCOS group and sub-groups were significantly different from unexplained and subgroups, and other and secondary other etiologies (P < 0.001).

### Gravidity

Naturally, healthy control showed significantly higher gravidity than the infertile group and subgroups (P < 0.001), while primary infertility had zero gravidity, and within the infertile group, subgroups PCOS and its groups had the lowest gravidity (P < 0.001).

### Parity

Healthy participants had significantly higher parity than the infertile group and subgroups (P < 0.001), primary infertile group had zero parity, while in the infertile cohort PCOS and subgroups exhibited the lowest parity (P < 0.001). Primary vs. secondary groups were also significantly different (P < 0.05).

### Disease Duration

Patients with unexplained infertility had the longest duration (P < 0.05), with other etiologies next, and PCOS individuals the shortest.

### Calcidiol

With the exception of primary other etiology cases, healthy controls exhibited significantly higher levels of calcidiol than all infertile groups (P < 0.001). However, the difference for other and secondary etiologies were less, with P< 0.01 and P< 0.05, respectively. Within the infertility subgroups only other and secondary etiologies vs. PCOS and primary PCOS were significantly different (P < 0.05). For all healthy and infertile groups the levels of calcidiol was not clinically severely deficient. For the controls, 16 (64%) were insufficient, 4 (16%) were normal, 2 (8%) deficient, and 3 (12%) had toxic levels (> 80 ng/mL). For the patients, over a half (44, 55%) were deficient, 30 (37.5%) insufficient, and 6 (7.5%) had normal levels. Over 92% of patients and 72% of controls exhibited insufficient/deficient levels.

### Calcitriol

With the exception of infertile primary other etiology cases, healthy controls exhibited higher levels of calcitriol than all other infertile groups (P < 0.001). Within the infertile subgroups, only other- and secondary other etiologies vs. PCOS and primary PCOS exhibited a significant difference (P < 0.05). At a cutoff of 100 pg/mL, 17 control (68%) but only 4 (5%) infertile women (specifically other etiologies) had normal levels.

### Direct Calcitriol/Calcidiol Ratio

Controls showed a higher ratio than all infertile subgroups (P < 0.001 overall, P < 0.05 vs secondary PCOS and primary unexplained, P < 0.01 vs. secondary unexplained, and no significant difference vs. primary other etiologies). Among infertile subgroups there were no significant differences. At a cutoff of 3.333 (100 pg/mL calcitriol vs. 30 ng/mL calcidiol), 64% (16) controls and 16.25% (13) infertile patients had normal ratios. Of the infertile cases, five were PCOS (3 primary, 2 secondary), five other etiologies (3 primary, 2 secondary), and four unexplained (all secondary).

Given that BMI is higher for the infertile patients than the controls, normalization of the levels of biomarkers for BMI (ng/mL / kg/m^2^) demonstrated greater levels of deficiency in the infertile group and subgroups for all markers.

Comparing the patients with primary vs. secondary causes showed nonsignificant difference due to the big differences among primary subgroups, and among secondary subgroups, for the three biomarkers.

### Mann-Whitney U test and ROC curve analysis of vitamin D parameters

**Figure 1:**
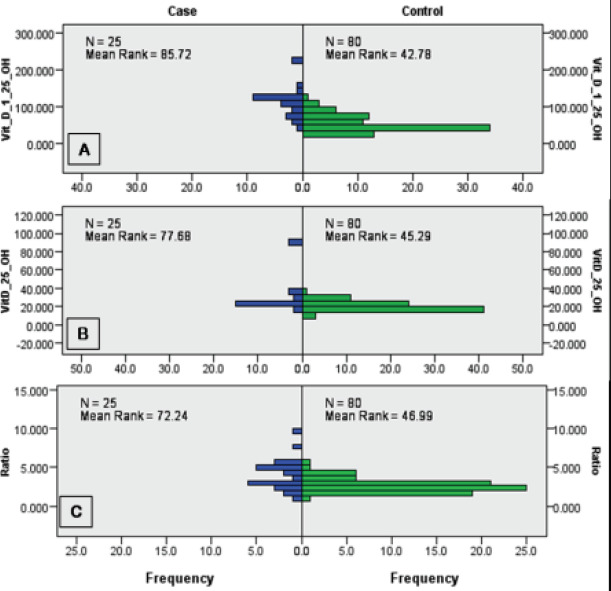
Mann-Whitney U test for cross tabulation of calcitriol, calcidiol and their ratio for infertile (n = 80) and fertile (n = 25) women. Significant differences noted across cases and controls for all biomarkers

**Figure 2:**
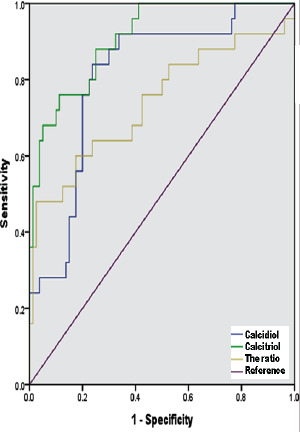
ROC curve analysis of AUC to determine ability of calcitriol, calcidiol, and their ratio to distinguish fertile (n = 25) from infertile (n = 80) women. Calcitriol exhibited the highest sensitivity and specificity; however calcidiol and the ratio were also predictive

BMI was the only normally distributed independent variable (P < 0.001). Using the Mann Whitney U test, data for calcidiol, calcitriol and their ratios were cross tabulated and identified a significant difference between cases and controls (2-sided Test asymptotic significance = 0.000; Figure 1). For categorical data, we used the Kruskal-Wallis test to cross-tabulate calcidiol, calcitriol and their ratio and found significant differences across the categories of P < 0.0001, <0.001, and < 0.040, respectively (Table 2). From ROC curve analysis, calcitriol was the best indicator of fertility AUC = 0.909 ± 0.031 (0.714-0.9.3), using the nonparametric approach, at asymptotic 95% CI (P < 0.001), followed by calcidiol AUC = 0.808 ± 0.048 (0.714-0.903); P < 0.001, and the ratio AUC = 0.740 ± 0.065 (0.612-0.869); P < 0.001 (Figure 2).

**Table 2:** Kruskal Wallis Test for cross tabulation of calcitriol, calcidiol and their ratio for infertile (n = 80) and fertile (n = 25) women, stratified for infertility. Data presented as mean ± standard deviation from the mean (SDM), median ± interquartile range (IQR), and P values

		Fertile controls	Polycystic ovary disease (n = 30)	Unexplained etiology (n = 23)	Other factors (n = 27)	P
Calcitriol	Mean ± SDM	113.99 ± 43.19	44.95 ± 17.5	48.50 ± 15.64	67.23 ± 28.26	<0.001
	Median ± IQR	119.80 ± 42.8	36.75 ± 20.4	42.40 ± 27	69.80 ± 50	
Calcidiol	Mean ± SDM	33.47 ± 22.07	17.31 ± 3.94	18.67 ± 2.82	24.89 ± 4.93	<0.001
	Median ± IQR	25.12 ± 6.36	16.35 ± 6.4	18.20 ± 4.6	24.73 ± 9.71	
Their Ratio	Mean ± SDM	4.09 ± 2.02	2.62 ± 0.86	2.61 ± 0.78	2.67 ± 0.94	0.040
	Median ± IQR	3.68 ± 2.49	2.36 ± 1.02	2.55 ± 0.99	2.67 ± 1.28	

## Results of the correlation analysis

### Healthy fertile control women

In healthy controls, the selected test characteristics were significantly positively correlated with each other. Vit D biomarkers exhibited a negative correlation with these characteristics, although was only significant for BMI vs. ratio. The ratio correlated negatively with calcidiol and positively with calcitriol (Table 3).

**Table 3:** Correlation of test characteristics in fertile women (n=25). Presented data are r/P values of non-parametric spearman correction analysis

	Gravidity	Parity	BMI	Calcidiol	Calcitriol	Ratio
Age	**0.614/0.0006**	**0.688/<0.0001**	**0.862/<0.0001**	0.047/0.413	-0.192/0.179	-0.302/0.071
Gravidity		**0.958/<0.0001**	**0.540/0.003**	-0.195/0.175	-0.045/0.416	-0.060/0.387
Parity			**0.586/0.001**	-0.203/0.166	-0.096/0.324	-0.021/0.461
BMI				0.146/0.243	-0.111/0.299	**-0.368/0.035**
Calcidiol					0.092/0.332	**-0.689/<0.0001**
Calcitriol						**0.540/0.0039**

### Infertile women

Infertile patient characteristics were significantly positively correlated with each other, with BMI significantly correlated with all characteristics. Calcidiol showed only significant positive correlation with gravidity, while BMI was nonsignificantly negatively correlated. Conversely, calcitriol was negatively associated with the characteristics, achieving significance vs. age and duration. The ratio exhibited a positive (nonsignificant) correlation with BMI, and negative association with the other characteristics. The ratio was positively correlated with calcidiol and calcitriol, with vs. calcitriol being significant (Table 4).

**Table 4:** Correlation between characteristics and Vit D biomarkers in infertile cases (n = 80). Data shown are r/P values of non-parametric Spearman correction analysis

	Gravidity	Parity	BMI	Duration	Calcidiol	Calcitriol	Ratio
Age	**0.579/<0.0001**	**0.701/<0.0001**	**-0.251/0.0122**	**0.776/<0.0001**	0.090/0.215	**-0.194/0.042**	**-0.376/0.0003**
Gravidity		**0.828/<0.0001**	**-0.322/0.002**	**0.311/0.003**	**0.252/0.012**	-0.033/0.385	**-0.224/0.023**
Parity			**-0.352/0.0007**	**0.441/<0.0001**	0.153/0.087	-0.129/0.126	**-0.311/0.003**
BMI				**-0.235/0.016**	-0.165/0.072	-0.029/0.400	0.141/0.106
Duration					0.0151/0.447	**-0.188/0.047**	**-0.304/0.003**
Calcidiol						**0.634/<0.0001**	0.0124/0.457
Calcitriol							**0.750/<0.0001**

### Infertile women with PCOS

Overall, fewer significant correlations were identified. Significant positively correlated data included, duration vs. age, parity and BMI; BMI vs. age; parity vs. age and gravidity. For biomarkers, calcitriol was significantly positively associated with only calcidiol and the ratio. (Table 5).

**Table 5:** PCOS-associated infertility patients (n = 30) and vitamin D biomarkers. Correlations are shown as r/P values of non-parametric Spearman correction analysis

	Gravidity	Parity	BMI	Duration	Calcidiol	Calcitriol	Ratio
Age	0.140/0.230	**0.454/0.006**	**0.576/0.0004**	**0.877/<0.0001**	0.040/0.416	-0.001/0.499	-0.180/0.171
Gravidity		**0.727/<0.0001**	-0.058/0.381	0.194/0.153	0.082/0.333	-0.069/0.360	-0.072/0.353
Parity			0.181/0.169	**0.474/0.004**	0.016/0.468	-0.129/0.248	-0.160/0.199
BMI				**0.443/0.007**	-0.055/0.387	0.001/0.498	-0.100/0.300
Duration					0.082/0.333	0.023/0.453	-0.165/0.191
Calcidiol						**0.635/<0.0001**	-0.112/0.2786
Calcitriol							**0.615/0.0002**

#### Infertile women with PCOS of primary causes (n = 21)

Significant positive correlations were observed for duration vs. age (r = 0.850; P < 0.0001), and vs. BMI (r = 0.443; P = 0.022); BMI vs. age (r = 0.593; P = 0.0023). For biomarkers, calcitriol was significantly positively associated with only calcidiol and the ratio (r = 0.518; P = 0.008, and r = 0.638; P = 0.009, respectively).

#### Infertile women with PCOS of secondary causes (n = 9)

Significant positive correlations were observed for duration vs. age (r = 0.836; P = 0.004), parity (r = 0.844; P = 0.016), and BMI (r = 0.647; P = 0.033). For biomarkers only calcitriol was significantly correlated with calcidiol (r = 0.820; P = 0.005).

Calcitriol

0.615/0.0002

### Infertile women with Unexplained Etiologies

Significant positive correlations were observed for age vs. gravidity, parity, and duration; gravidity vs. parity, and BMI; and parity vs. BMI. For biomarkers, calcidiol was significantly positive vs. calcitriol and BMI. Calcitriol significantly correlated negatively vs. age and duration, and positively vs. ratio. Ratio showed similar correlations vs. age and duration (Table 6).

**Table 6:** Infertility of unexplained etiology (n = 23) and vitamin D biomarkers. Correlations are shown as r/P values of non-parametric Spearman correction analysis

	Gravidity	Parity	BMI	Duration	Calcidiol	Calcitriol	Ratio
Age	**0.620/0.0008**	**0.612/0.0009**	0.058/0.397	**0.779/<0.0001**	0.036/0.435	**-0.403/0.028**	**-0.415/0.025**
Gravidity		**0.875/<0.0001**	**0.450/0.016**	0.169/0.220	0.130/0.277	-0.060/0.393	-0.150/0.247
Parity			**0.447/0.016**	0.164/0.227	0.116/0.299	-0.117/0.298	-0.181/0.205
BMI				-0.231/0.145	**0.535/0.004**	0.167/0.223	-0.115/0.300
Duration					-0.160/0.233	**-0.566/0.0024**	**-0.471/0.012**
Calcidiol						**0.459/0.014**	-0.171/0.218
Calcitriol							**0.770/<0.0001**

#### Infertile women with primary Unexplained Etiologies (n = 7)

Significant positive correlation was observed only for duration vs. age (r = 0.873; P < 0.010), and calcidiol vs. BMI (r = 0.883; P = 0.008).

#### Infertile women with secondary Unexplained Etiologies (n = 16)

Significant positive correlations were observed for age vs. duration (r = 0.801; P = 0.0002), gravidity vs. parity (r = 0.735; P = 0.0008). For biomarkers, significant negative correlations were noted for calcitriol and the ratio vs. age (r = -0.640; P = 0.0045; r = -0.771; P = 0.0004) and duration (r = -0.638; P = 0.0046; r = -0.683; P = 0.0023). While calcitriol correlated positively with the ratio (r = 0.849; P < 0.0001).

#### Infertile women with Other Etiologies

Significant positive correlations were noted for age vs. gravidity, parity and duration; gravidity vs. parity, and BMI; and parity vs. BMI. For biomarkers, significant negative correlations were noted for calcidiol vs. duration; calcitriol vs. all characteristic, except with BMI which was positive. Inter-biomarker relationships showed positive correlation for calcidiol vs. calcitriol, and calcitriol vs. ratio. The ratio showed negative correlation with age, gravidity and parity, but positive vs. BMI (Table 7).

**Table 7:** Other etiology infertility cases (n = 27) correlation with vitamin D biomarkers, described as r/P values of non-parametric Spearman correction analysis

	Gravidity	Parity	BMI	Duration	Calcidiol	Calcitriol	Ratio
Age	**0.685/<0.0001**	**0.812/<0.0001**	-0.441/0.0106	**0.665/<0.0001**	-0.24/0.114	**-0.599/0.0005**	**-0.595/0.0005**
Gravidity		**0.788/<0.0001**	**-0.378/<0.026**	0.304/0.062	-0.061/0.381	**-0.503/<0.004**	**-0.519/<0.003**
Parity			**-0.551/<0.002**	**0.471/<0.007**	-0.123/0.271	**-0.600/0.0005**	**-0.631/0.0002**
BMI				-0.209/0.148	0.130/0.259	**0.628/0.0002**	**0.692/<0.0001**
Duration					**-0.363/0.031**	**-0.389/0.022**	-0.288/0.073
Calcidiol						**0.599/0.0005**	0.190/0.172
Calcitriol							**0.871/<0.0001**

#### Infertile women with primary Other Etiologies (n = 7)

Significant positive correlations were observed only for BMI vs. calcitriol and vs. ratio (r = 0.786; P = 0.024 for both), and calcitriol and ratio (r = 0.893; P = 0.006).

#### Infertile women with secondary Other Etiologies (n = 20)

Significant positive correlations were observed for age vs. gravidity and BMI (r = 0.845; P < 0.0001; r = 0.731; P = 0.030, respectively); gravidity vs. parity and duration (r = 0.719; P = 0.0002; r = 0.476; P = 0.017, respectively); parity vs. duration (r = 0.627; P < 0.002). Negative correlations were noted for age vs. parity and duration (r = -0.426; P < 0.0001; r = -0.382; P = 0.0001, respectively); gravidity vs. BMI (r = -0.407; P = 0.037); and parity vs. BMI (r = -0.603; P < 0.003). For biomarkers and characteristics, significant negative correlations were noted for calcidiol vs. duration (r = -0.462; P = 0.020); calcitriol vs. all (except BMI) (r = -0.598; P = 0.0006 with age, r = -0.560; P = 0.005 with gravidity, r = -0.693; P = 0.0004 with parity, and r = -0.588; P = 0.003 with duration); ratio vs. gravidity (r = -0.616; P < 0.002), parity (r = -0.740; P < 0.0001), and duration (r = -0.434; P = 0.028). Positive correlations were observed for calcitriol vs. BMI (r = 0.529; P = 0.008); ratio vs. age (r = 0.845; P < 0.003) and BMI (r = 0.617; P < 0.002). Inter-biomarker analysis revealed positive associations for calcidiol vs. calcitriol (r = 0.599; P = 0.0005); calcitriol vs. ratio (r = 0.871; P < 0.0001).

## Discussion

While our study is not unique, it offers new insight into infertility and association with vitamin D in our area, as Vit D levels in reproductive-aged Aljouf Saudi women have not been published. It also shows the applicability of the biomarkers. We show that Vit D is insufficient/deficient in 82% of healthy participants, while cases expressed a rate of 90 – 100%. We found that calcitriol was most sensitive in differentiating between these groups.

We describe the situation where calcidiol is the prohormone and calcitriol the active form, in which calcitriol is the more precise discriminant factor 26. Similar to the current literature, including meta-analyses 6,25,26,43-47, we found wide-spread Vit D deficiency/insufficiency among healthy controls, and significantly worse scenarios in infertile women. While the three biomarkers exhibited significant difference between controls and cases, we found calcitriol > ratio > calcidiol for discrimination. For individual disease categories, we found biomarker performance was worse for PCOS, then unexplained- and other etiologies. On the whole, a positive correlation was observed for healthy control characteristics, while only the ratio was negatively correlated with BMI. Similarly for infertile patients, positive correlations were observed for all with the exception of BMI which was negatively correlated with the three biomarkers and calcidiol vs gravidity which was positively correlated.

All of the other etiology infertility patients (cervical, uterine, tubal or peritoneal origin or anovulation) exhibited low Vit D levels. Low Vit D is significantly correlated with endometriosis risk and severity, as well as a number of other gynecological conditions including leiomyoma, uterine myoma, and dysmenorrhea 30,34,48-54. For severe cases of endometriosis, calcidiol levels were half of those in healthy controls or in women with less severe endometriosis. Contrary to our findings, calcidiol was positively associated with gravidity and parity 55, while we noted negative associations between both of calcitriol and ratio vs. gravidity and parity. Some in vitro research noted an effect of calcitriol on reduction of inflammatory markers and factors associated with endometrial stroma cells 49, others purported that the data is insufficient to definitively indicate a Vit D-endometriosis relationship 31,56,57. However, we do know that estradiol and progesterone are inversely correlated with calcidiol levels 58. Endometriosis patients showed significantly higher calcidiol levels but calcitriol was nonsignificantly different from healthy controls 49,59. Calcidiol exhibits significant seasonal variation while calcitriol does not 60-62.

The pathogenesis of endometriosis may be associated with high levels of Vit D resulting in impaired elimination of endometrial cells in the peritoneal cavity 63. Our findings were contrary to these, we noted low calcidiol and consistent and lower levels of calcitriol in the other etiology patients, and calcidiol correlated negatively with only the duration, while calcitriol and the ratio were negatively correlated with all characteristics except BMI. This may be related to a “change point issue”, wherein serum and follicular fluid levels of AMH are negatively correlated with Vit D to ~30 ng/mL and thereafter show a positive insignificant relationship with tubal factor infertility patients. Further, significant negative seasonal variations were noted for both 61, which may be indicative of the critical nature of lower rather than higher Vit D levels. The impact of Vit D in IVF and associated parameters including resulting pregnancy is controversial 28. However, investigating IVF outcomes in an egg-donor model, which separates the effect of Vit D on eggs and endometrium, data indicates that Vit D effect may occur via the endometrium. Deficiency and insufficiency levels of Vit D exhibited similar negative consequences 64.

A study of Iranian women reported similar data to our PCOS cases, whereby the lowest Vit D levels were noted but did not exhibit any correlation with patient characteristics (or between the biomarkers). Vit D levels in serum and follicular fluid were lower in PCOS patients compared to controls and correlated negatively with BMI. Vit D controls insulin expression and sensitivity, and in turn α1-hydoxylase expression and calcitriol level 65. Low levels of Vit D were linked to both PCOS and associated risk factors such as obesity and insulin resistance 31,37,45,66. In the Iranian study, lower Vit D was noted among PCOS-infertile women, specifically those who were obese, than controls 67. Ovulation is significantly improved in PCOS cases where low Vit D is treated 68,69, and cases with higher serum and follicular levels had significantly increased IVF pregnancy rate than others 70. Later in the reproductive period, Vit D and ovarian reserve are significantly positively correlated 71. Ovarian stimulation outcome in PCOS is significantly associated with Vit D deficiency 72. PCOS patients show a negative correlation with Vit D vs testosterone, SHBG, free androgen index, DHEAS levels, and LH/FSH ratio, which is worse in obese patients^73^,^74^.

However, pregnancy outcome was not significantly associated with Vit D levels 75. Post-confounder normalization, PCOS patients showed association with higher Vit D levels; however, this did not lead to improved metabolic function 62. Serum Vit D was not associated with PCOS or AMH 76, and there was no significant correlation between conceiving or miscarriage risk with pre-pregnancy Vit D levels 3. These studies may be confounded by the fact that both patients and controls exhibit insufficient/deficient levels of Vit D^62^, or that a low cutoff level (20 ng/mL) is used 6,76-78. This is a critical point as the recommended reproductive thresholds for serum calcidiol is higher (≥ 45 ng/mL) than for the non-pregnant population 64,79.

Our study found a significant reduction in Vit D in unexplained/known etiology cases, with positive correlation noted between calcidiol vs. BMI, and negative between calcitriol/ratio vs. duration and age. Contrary to this, there was no noted association between Vit D deficiency and ovarian stimulation outcome 72. However, in one African study, incorporating all etiologies, with a major unexplained, infertile patients did not exhibit lower Vit D than the controls 80. Nor was correlation observed with Vit D and follicular count/AMH^77^,^78^. While follicular and serum Vit D levels are putative markers of egg and embryo quality, and higher levels result in higher pregnancy chance, a positive association has only been observed and no significant correlations reported 81,82.

Given the seasonal variation in calcidiol, we assessed the efficacy of calcidiol, calcitriol and their ratio as predictive markers for infertility vs. healthy controls. Calcidiol was inferior to calcitriol in predicting infertility and negatively correlated with the clinical characteristics of all studies etiologies. This study provides insight into the variation of these three makers as they relate to infertility. In summary, the lowest rate of Vit D deficiency was in PCOS cases and is similar to unexplained cases, while other etiology preformed marginally better. Calcidiol showed negative correlation with BMI in patients, while calcitriol and ratio exhibited a positive correlation with BMI of healthy controls. All biomarkers were negatively correlated with disease duration in all patients. Consequently, it is reasonable to predict an association for Vit D deficiency and unexplained factor infertility. The notion of nonsignificant differences for the three biomarkers comparing the primary vs. the secondary causes of infertility in this study, due to the big difference among subgroups of each, was previously reported 6.

The study was limited by the small case numbers, especially in the subgroups, a year round sample collection period could have allowed seasonal variation, and, since we did not estimate reproductive hormones levels, we did not analyze their correlations with Vit D biomarkers.

## Conclusion

Over 90% of the infertile women in the study exhibited Vit D deficit as determined by the three biomarkers. Lowest levels were reported for PCOS and unexplained reasons, and then other etiologies. Calcitriol was the optimal predictor of infertility, and the ratio was discriminatory for healthy vs. infertile participants. Given the seasonal nature of calcidiol, we recommend the use of calcitriol and their ratio as the more precise markers. It is vital that standardized no-skeletal Vit D levels are fully characterized in the female reproductive-aged populations. Our data corroborate a role for Vit D in unexplained infertility, and indicate a therapeutic role for Vit D supplementation.
